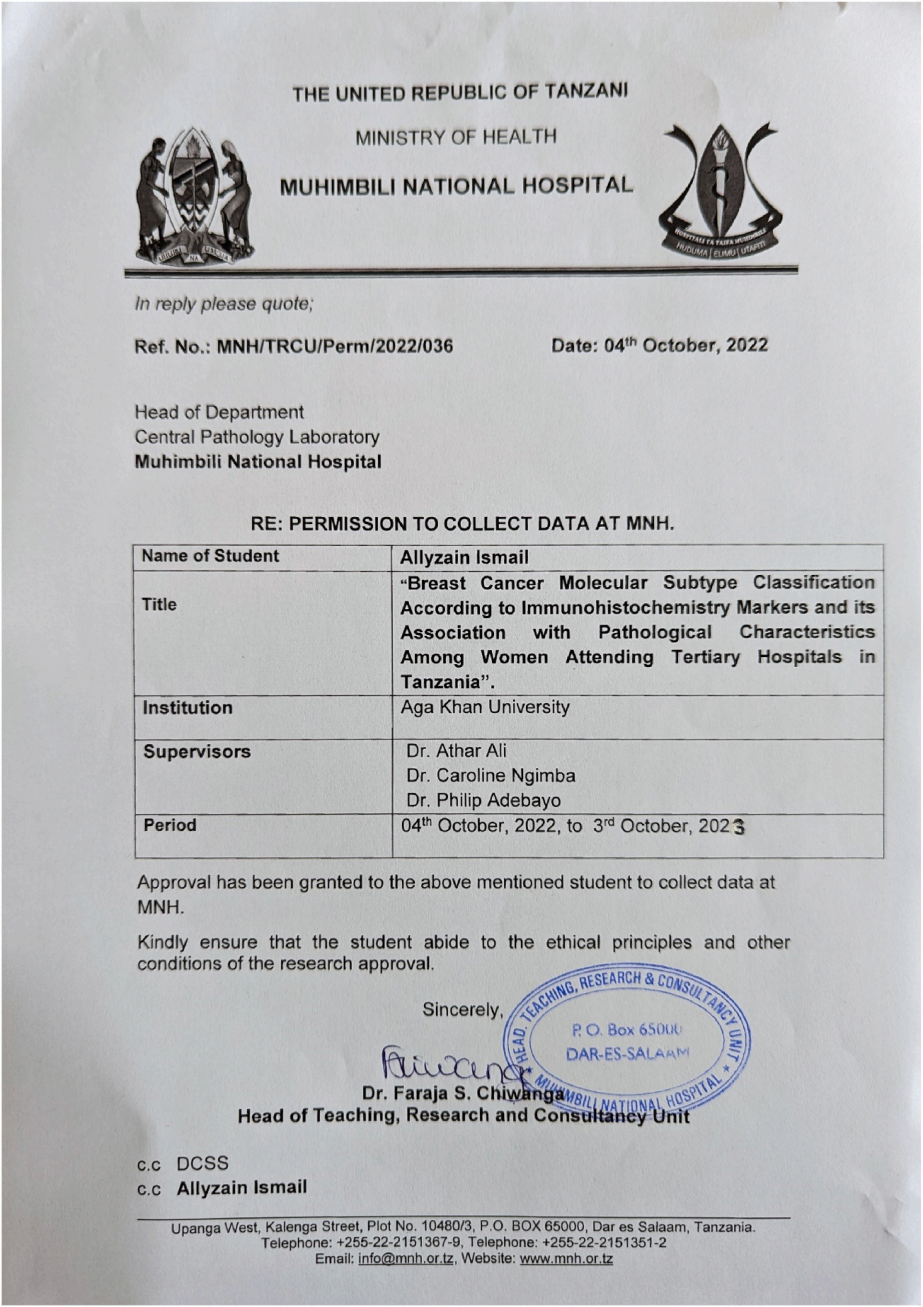# Breast cancer molecular subtype classification according to immunohistochemistry markers and its association with pathological characteristics among women attending tertiary hospitals in Tanzania

**DOI:** 10.1016/j.heliyon.2024.e38493

**Published:** 2024-09-26

**Authors:** Allyzain Ismail, Sajida Panjwani, Neelam Ismail, Caroline Ngimba, Innocent Mosha, Philip Adebayo, Ally Mwanga, Ali Akbar Zehri, Aidan Njau, Ali Athar

**Affiliations:** a Department of Surgery, Aga Khan University Medical College, Dar es Salaam, Tanzania; b Department of Family Medicine, Aga Khan University Medical College, Dar es Salaam, Tanzania; c Department of Pathology, Aga Khan Hospital, Dar es Salaam, Tanzania; d Department of Pathology, Muhimbili National Hospital, Dar es Salaam, Tanzania; e Department of Medicine, Aga Khan University Medical College, Dar es Salaam, Tanzania; f Department of Surgery, Muhimbili National Hospital, Dar es Salaam, Tanzania

**Keywords:** Breast cancer, Immunohistochemistry, Molecular subtype, Tanzania, Africa

## Abstract

**Background:**

Breast cancer immunohistochemistry is a biological characteristic of the tumour which has a role to diagnose molecular subtype, prognosticate and guide treatment and is categorised into 4 subtypes. Data in Tanzania was lacking and was based off data extrapolated from studies in Western Africa thus hypothesizing that women of African ancestry predominately develop Triple Negative Breast Cancer (TNBC).

**Methods:**

A retrospective cross-sectional study was carried out at two tertiary referral hospitals on participants who were recruited from the cancer registries from 2015 to 2022. Prevalence of each molecular subtype was determined and association between molecular subtype to demographic and pathological characteristics were evaluated. Predictors of molecular subtypes was then determined using logistic regression.

**Results:**

Total number of participants were 1214, median age was 50 (IQR: 41–61), median tumor size was 5 cm (IQR: 4–7) with lymph node positivity in 73.7 %. Immunohistochemistry studies showed estrogen, progesterone and Human Epidermal Growth Factor Receptor 2 (HER2) receptor positivity in 54.4 %, 34.4 % and 27.8 % of cases respectively. Molecular subtype classification prevalence for Luminal A was 21.17 % (95 % CI: 18.87–23.47), for Luminal B 35.75 % (95 % CI: 33.05–38.45), for HER2 enriched 11.86 % (95 % CI: 10.04–13.68) and for TNBC 31.22 % (95 % CI: 28.61–33.83). Significant association was seen between molecular subtype with age, tumor size, tumor grade and lymph node involvement. Predictors of Luminal tumors were larger tumor size (aOR 1.217, 95 % CI: 1.149–1.291) no lymph node involvement (aOR 0.429, 95 % CI: 0.313–0.589) while an advanced tumor grade reduced likelihood (aOR 0.041, 95 % CI: 0.011–0.019).

**Conclusion:**

In Tanzania Luminal B was most predominant subtype presenting at an earlier age and associated with more favorable pathological characteristics.

## Highlights

• Prevalence of breast cancer molecular subtypes according to St Gallen International Breast Cancer Conference.
• Predominance of Luminal type breast cancer.
• Favorable pathological characteristics with luminal type breast tumors.

## Abbreviations

AKH: Aga Khan Hospital
aOR: Adjusted Odds Ratio
ASCO/CAP: American Society of Clinical Oncology/College of American Pathologists
CI: Confidence Intervals
ER: Estrogen Receptor
FISH: Fluorescent In Situ Hybridisation
HER2: Human Epidermal Growth Factor Receptor 2
IDC NST: Invasive Ductal Carcinoma of No Specific Type
IHC: Immunohistochemistry
IQR: Inter Quartile Range
MNH: Muhimbili National Hospital
OR: Odds Ratio
PR: Progesterone Receptor
TNBC: Triple Negative Breast Cancer

## 1 Introduction

Breast cancer is the most common malignancy among women worldwide and the second most prevalent among females in sub-Saharan Africa [1,2]. Treatment is a multidisciplinary approach consisting of regional control, via surgery and radiation, and systemic medical therapy through endocrine, chemotherapy and targeted biological therapy. These therapy strategies are determined by the molecular subtype of the breast cancer which can be established via immunohistochemistry (IHC) analysis for molecular markers [3]. Immunohistochemistry plays a role as both a prognostic factor as well as guidance for treatment [4]. It involves the use of antibodies to detect specific antigens expressed on a tumour [5].

Initially, at the 12th International Breast Cancer Conference held at St Gallen, breast cancer was divided into 4 molecular subtypes depending on their Estrogen Receptor (ER) and Progesterone Receptor (PR) positivity, presence of overexpression of the Human Epidermal Growth Factor Receptor 2 (HER2) and nuclear protein Ki67 [6]. These subtypes are Luminal A, Luminal B, HER2 enriched and Triple Negative Breast Cancer (TNBC) of which each subtype has its own management algorithm. Subsequent conferences fine-tuned the definition criteria for each molecular subtype.

Pathological characteristics of a tumour is concerned with changes in tissue morphology and architecture which are characteristic for the disease. Multiple pathological characteristics have been studied to guide treatment and help prognosticate the condition with regards to disease morbidity and mortality [7]. Among these characteristics, tumour size, tumour grade and lymph node involvement have been extensively studied and seen to be more favorable in hormone receptor positive tumors while less favorable in TNBC [8]. Therefore, IHC findings and pathological characteristics have been used hand in hand to establish guidelines.

Molecular subtypes of breast cancer have shown variation between different regions of the world [[9], [10], [11], [12], [13]]. Similarly, within Africa, Northern African countries have a tendency towards hormonal positivity whereas TNBC was more common in Western Africa [[14], [15], [16], [17], [18]]. However, data on IHC patterns in Tanzania is lacking with data extrapolated from studies in Western Africa as well as on African American women hypothesize that women of African ancestry predominately develop TNBC [19]. Studies however carried out in Kenya challenge this notion with Luminal A or B subtype taking predominance [20,21]. Table 1 is a summary of the studies carried out on prevalence of molecular subtypes of breast cancer around the world.

**Table 1:** Variation in prevalence of molecular subtype of breast cancer.

Region	Author	Luminal A (%)	Luminal B (%)	HER2^a^ enriched (%)	TNBC^b^ (%)	Hormonal positivity (%)
Germany	Hennigs (2016)	44.7	38	5	12.3	82.7
Macedonia	Kondov (2018)	26.6	55.5	8.6	9.3	82.1
India	Ambroise (2011)	12	36	27	25	48
Korea	Park (2012)	53.1	21.7	9	16.2	74.8
Saudi Arabia	Alnegheimish (2016)	58.5	14.5	12.2	14.8	73
Nigeria	Wemimo (2023)	16.8	20.4	20.3	42.5	37.2
Angola	Miguel (2017)	25.7	27.2	15.7	31.4	52.9
Morocco	Errahhali (2017)	61.1	16.1	8.6	14.2	77.2
Kenya	Sayed (2021)	34.8	35.8	10.7	18.7	70.6

a Human Epidermal Growth Factor Receptor 2.

b Triple Negative Breast Cancer.

Establishing the prevalence of each subtype may guide institutions to understand the needs of the population so as to increase availability of the required care, especially in resource limited settings such as ours, with regards to molecular subtype so as to address peri-surgical care. As such knowledge within the Tanzanian population was lacking the objective of this study was to determine the prevalence of each breast cancer molecular subtype and establish their association to demographic and pathological characteristics.

## 2 Methodology

### 2.1 Study setting

A retrospective cross-sectional study was carried out at Muhimbili National Hospital (MNH) and Aga Khan Hospital (AKH) which are 2 of the 3 centres which offer IHC testing in the country. The third being Kilimanjaro Christian Medical Centre whose focus serves residents of North-Western region only. MNH and AKH are a public and private institute respectively which offer IHC staining on tissue blocks within their in-hospital laboratories. Both centres receive referrals from all over Tanzania, AKH from its country wide outreach centres and MNH as the national referral centre. A study evaluated ER IHC performed at MNH as compared to University of California, San Francisco and showed excellent concordance hence exemplifying their laboratory capacity while the AKH laboratory is accredited by Southern African Development Community Accreditation Service [22].

### 2.2 Study population

There was a total of 3017 entries within the hospital breast cancer registries between 2015 and 2022 at the two centres. Only patients with a breast tissue biopsy confirming primary breast cancer which had undergone further IHC analysis were included and those with inadequate or missing reports were excluded. Via convenient sampling technique all 3000 charts were stratified by date of entry and those eligible based off inclusion and exclusion criteria were evaluated with a total a number of participants studied at 1214.

### 2.3 Variables

Data was collected with participants identified from cancer registries after which their respective histopathology reports retrieved and collected via a data collection table which was created based off other studies that looked at similar variables (Appendix I). As per the American Society of Clinical Oncology/College of American Pathologists (ASCO/CAP) guidelines ER and PR status was considered positive if ≥ 1 % nuclei of tumour cells were stained [23]. The HER2 status was determined by the protein expression on tumour cell membranes and scored as 0, 1+, 2+ and 3+ as per ASCO/CAP guidelines whereby ≤1+ was considered HER2 negative, 2+ was considered HER2 equivocal and 3+ was considered HER2 positive [24]. Equivocal results that had undergone further Fluorescent In Situ Hybridisation (FISH) testing was used to ascertain positivity otherwise equivocal reports lacking FISH tests were considered void and not included in the study. Luminal A, Luminal B, HER2 enriched or TNBC was determined as per the 13th International Breast Cancer Conference definition criteria (Table 2).

**Table 2:** Molecular subtype according to the 13th International Breast Cancer Conference.

Molecular subtype		ER	PR	HER2
Luminal A		+	+/−	–
Luminal B	HER2^a^ negative	+	+/−(Ki-67 > 20 %)	–
	HER2^a^ positive	+	+/−(Any Ki-67 level)	+
HER2^a^ enriched		–	–	+
TNBC^b^		–	–	–

a Human Epidermal Growth Factor Receptor 2.

b Triple Negative Breast Cancer.

### 2.4 Analysis

Data was analysed using Statistical Package for the Social Sciences version 25 whereby initially normality of continuous data was assessed and presented as medians with interquartile ranges (IQR) whereas categorical data were expressed in frequencies and percentages. Variables were assessed for association with molecular subtypes using Pearson's chi-square and Kruskal–Wallis with a p-value <0.05 considered statistically significant. Significantly associated variables were entered into logistic regression models for true association with a Hosmer-Lemeshow test used to evaluate the model as fit. Odds ratio (OR) with corresponding 95 % confidence intervals (CI) and p-values were reported.

### 2.5 Ethical considerations

The study was performed after ethical approval by the Ethical Review Committee of Aga Khan University (IED/2022/009/fb/016) and Muhimbili National Hospital (MNH/TRCU/Perm/2022/036) in line with the university policies, laws and regulation and ethical guidelines.

## 3 Results

**Fig. 1:**
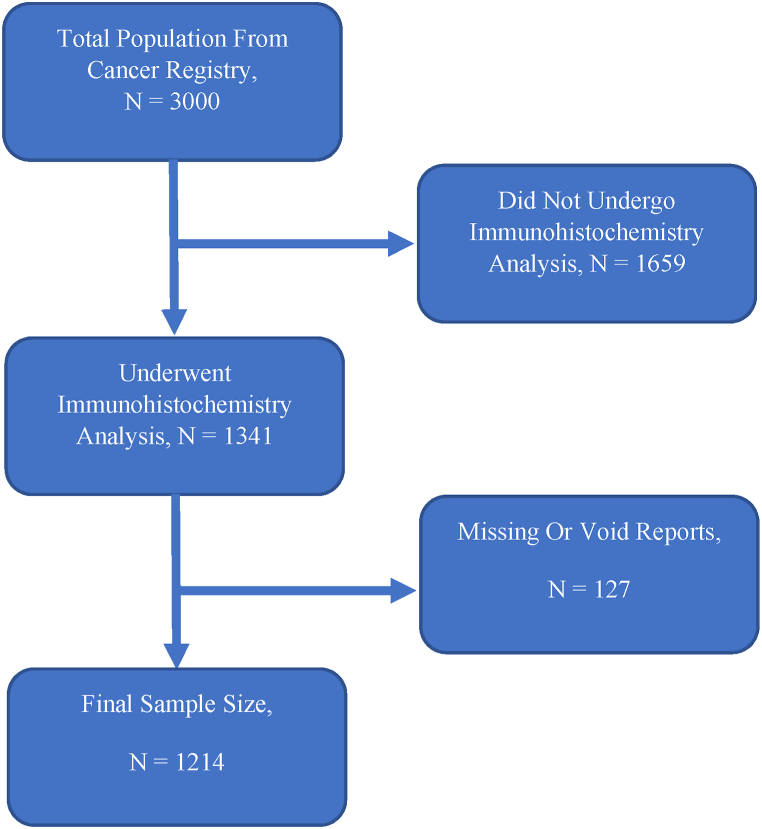
Final sample size flowchart.

The demographic and pathological characteristics of recruited participants are presented in Table 3. From 2015 to 2022, the total number of participants included in the study was 1214 (Fig. 1) with 1156 (95.2 %) from MNH. The median age was 50 (IQR: 41–61) with an age range of 18–89 years with majority (45.1 %) in the menopausal age group of 40–55 years of age, 685 (56.4 %) were from Dar-es-Salaam and 97.8 % of African ethnicity. The median tumor size was 5 cm (IQR: 4–7), of which majority (n = 585, 48.2 %) of samples were more than 5 cm in largest dimension. The predominant histological subtype was Invasive Ductal Carcinoma of No Special Type (IDC NST) at 89.7 % with the most common pathological grade according to the Nottingham classification as grade 2 (74.8 %) and lymph node positivity in 895 (73.7 %) of the cases.

**Table 3:** Demographic and pathological characteristics of recruited entries (N = 1214).

Variables	Number of patients
Age in years	50 (41–61)^a^
Age specific groups, n (%)	
< 40 years	232 (19.1)
40–55 years	547 (45.1)
> 55 years	435 (35.8)
Ethnicity, n (%)	
African	1187 (97.8)
Arab	10 (0.8)
Indian	15 (1.2)
European	2 (0.2)
Hospital, n (%)	
Aga Khan Hospital, Dar es Salaam	58 (4.8)
Muhimbili National Hospital	1156 (95.2)
Region of origin, n (%)	
Arusha	26 (2.1)
Dodoma	27 (2.2)
Dar es Salaam	685 (56.4)
Morogoro	74 (6.1)
Mwanza	24 (2)
Zanzibar	15 (1.2)
Other	363 (29.9)
Histological subtype, n (%)	
IDC NST^b^	1089 (89.7)
Invasive Lobular	41 (3.4)
Papillary	22 (1.8)
Mucinous	26 (2.1)
Medullary	21 (1.7)
Other	15 (1.2)
Tumor size, largest dimension in cm	5 (4–7)^a^
Size specific groups, n (%)	
< 2 cm	114 (9.4)
2 – 5 cm	515 (42.4)
> 5 cm	585 (48.2)
Nottingham tumor grade, n (%)	
I	53 (4.4)
II	908 (74.8)
III	253 (20.8)
Lymph node involvement, n (%)	
No	319 (26.3)
Yes	895 (73.7)

a Median (IQR).

b Invasive Ductal Carcinoma of No Special Type.

**Fig. 2:**
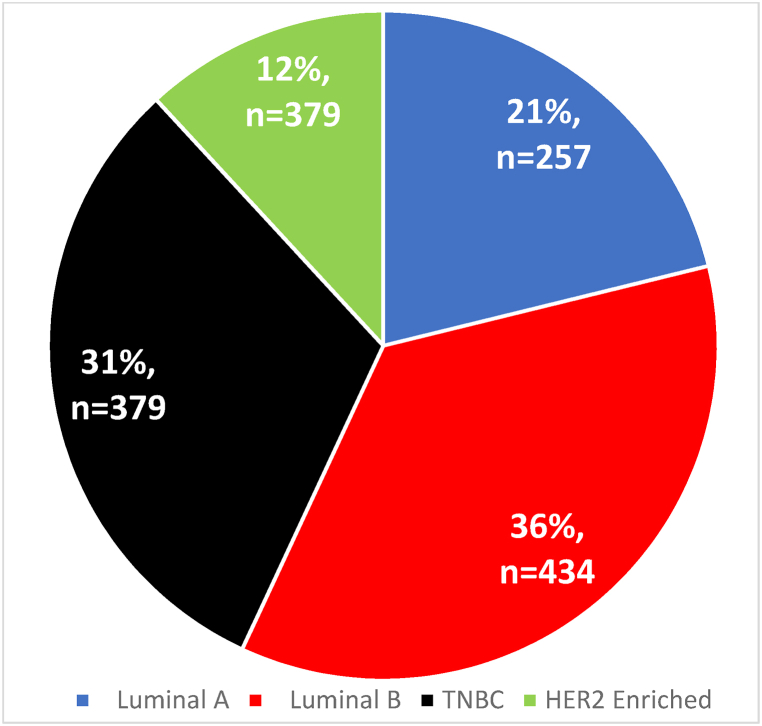
Distribution of breast cancer molecular subtypes.

Immunohistochemistry studies showed ER positivity in 660 (54.4 %) cases, PR positivity in 418 (34.4 %) cases and HER2 positivity in 338 (27.8 %) of cases with majority (56.9 %) expressing at least ER or PR hormonal receptor positivity. Therefore, the molecular subtype classification prevalence for Luminal A was 21.17 % (95 % CI: 18.87–23.47), for Luminal B was 35.75 % (95 % CI: 33.05–38.45), for HER2 enriched was 11.86 % (95 % CI: 10.04–13.68) and for TNBC was 31.22 % (95 % CI: 28.61–33.83) (Fig. 2). When molecular subtype was compared according to if the patient was from public or private hospital no significant association was seen (p = 0.638) with a similar distribution pattern noted. Table 4 shows the distribution of IHC receptor markers and their grouping into molecular subtyping.

**Table 4:** Distribution of immunohistochemistry receptor markers and molecular subtypes at public and private hospital.

Variables	Total number of patients (%)	Public hospital, n (%)	Private Hospital n (%)	P - Value
ER^a^ status				
Positive	660 (54.4)	624 (54)	36 (62.1)	0.227
Negative	554 (45.6)	532 (46)	22 (37.9)	
PR^b^ status				
Positive	418 (34.4)	391 (33.8)	27 (46.6)	0.051
Negative	796 (65.6)	765 (66.2)	31 (53.4)	
HER2^c^ status				
Positive	338 (27.8)	325 (28.1)	13 (22.4)	0.345
Negative	876 (72.2)	831 (71.9)	45 (77.6)	
Presence of hormonal receptor				
Positive	691 (56.9)	654 (56.6)	37 (63.8)	0.279
Negative	523 (43.1)	502 (43.4)	21 (36.2)	
Molecular subtype				
Luminal A	257 (21.2)	242 (20.9)	15 (25.9)	0.638
Luminal B	434 (35.7)	412 (35.6)	22 (37.9)	
HER2 enriched	144 (11.9)	137 (11.9)	7 (12.1)	
TNBC^d^	379 (31.2)	365 (31.6)	14 (24.1)	

a Estrogen Receptor.

b Progesterone Receptor.

c Human Epidermal Growth Factor Receptor 2.

d Triple Negative Breast Cancer.

Patients with Luminal A tumors had a median age of 52 years (IQR: 43–62) with 89.9 % being IDC NST, lymph node positivity in 70 %, grade 2 in 81.3 % and a median tumor size of 5.5 cm (IQR: 4–7). Luminal B tumors had a median age of 49 years (IQR: 40–60) with 88.2 % being IDC NST, lymph node positivity in 64.1 %, grade 2 in 91 % and a median tumor size of 6 cm (IQR: 4–7). The HER2 enriched tumors had a median age of 48 years (IQR: 40–58) with 90.3 % being IDC NST, lymph node positivity in 81.3 %, grade 2 in 71.5 % and a median tumor size of 6 cm (IQR: 5–7). The TNBC tumors had a median age of 51 (IQR: 41–64) with 91 % being IDC NST, lymph node positivity in 84.4 %, grade 2 in 53 % and a median tumor size of 5 cm (IQR: 3–6). A Chi-Square and Kruskal Wallis test performed on categorical and non-parametric continuous variables respectively revealed a significant association (p-value <0.05) between molecular subtype with age, tumor size, tumor grade and lymph node involvement but no association seen with ethnicity, region of origin, public or private hospital and histological subtype. The distribution of variables according to molecular subtype and their associations are summarized in Table 5.

**Table 5:** Association of demographic and pathologic characteristics with breast cancer molecular subtypes.

Variable	Molecular Subtype				P – value
	Luminal A N = 257	Luminal B N = 434	HER2 Enriched N = 144	TNBC N = 379	
Age in years	52 (43–62)^a^	49 (40–60)^a^	48 (40–58)^a^	51 (41–64)^a^	0.013^b^
Age specific groups, n (%)					
< 40 years	35 (13.6)	93 (21.4)	26 (18.1)	78 (20.6)	0.004
40–55 years	121 (47.1)	202 (46.5)	77 (53.5)	147 (38.8)	
> 55 years	101 (39.3)	139 (32)	41 (28.5)	154 (40.6)	
Ethnicity, n (%)					
African	251 (97.7)	421 (97)	140 (97.2)	375 (98.9)	0.286
Other	6 (2.3)	13 (3)	4 (2.8)	4 (1.1)	
Hospital, n (%)					
Aga Khan Hospital, Dar es Salaam	15 (25.9)	22 (37.9)	7 (12.1)	14 (24.1)	0.638
Muhimbili National Hospital	242 (20.9)	412 (35.6)	137 (11.9)	365 (31.6)	
Region of origin, n (%)					
Arusha	5 (1.9)	14 (3.2)	4 (2.8)	3 (0.8)	0.273
Dodoma	7 (2.7)	8 (1.8)	3 (2.1)	9 (2.4)	
Dar es Salaam	147 (57.2)	231 (53.2)	76 (52.8)	231 (60.9)	
Morogoro	11 (4.3)	26 (6)	12 (8.3)	25 (6.6)	
Other	87 (33.9)	155 (35.7)	49 (34)	111 (29.3)	
Histological subtype, n (%)					
IDC NST^c^	231 (89.9)	383 (88.2)	130 (90.3)	345 (91)	0.119
Invasive Lobular	6 (2.3)	18 (4.1)	9 (6.3)	8 (2.1)	
Other	20 (7.8)	33 (7.6)	5 (3.5)	26 (6.9)	
Tumor size in cm	5.5 (4–7)^a^	6 (4–7)^a^	6 (5–7)^a^	5 (3–6)^a^	0.001^b^
Size specific groups, n (%)					
< 2 cm	25 (9.7)	38 (8.8)	7 (4.9)	44 (11.6)	0.001
2 – 5 cm	103 (40.1)	171(39.4)	42 (29.2)	199 (52.5)	
> 5 cm	129 (50.2)	225 (51.8)	95 (66)	136 (35.9)	
Nottingham tumor grade, n (%)					
I	34 (13.2)	17 (3.9)	1 (0.7)	1 (0.3)	0.001
II	209 (81.3)	395 (91)	103 (71.5)	201 (53)	
III	14 (5.4)	22 (5.1)	40 (27.8)	177 (46.7)	
Lymph node involvement, n (%)					
No	77 (30)	156 (35.9)	27 (18.8)	59 (15.6)	0.001
Yes	180 (70)	278 (64.1)	117 (81.3)	320 (84.4)	

a Median (IQR).

b Kruskal Wallis Test.

c Invasive Ductal Carcinoma of No Special Type.

Separate binary logistic regression models were run for each molecular subtype whereby significantly associated variables were inserted into the model to ascertain their effect on the likelihood of each molecular subtype (Table 6). The model was considered fit on each occasion with a Hosmer and Lemeshow test of p-value >0.05 in each instance. Luminal A tumors were significantly associated with tumor size and tumor grade. They had a slightly increased odds with regards to tumor size hence larger tumors were more likely to be Luminal A tumors (Adjusted Odds Ratio (aOR) 1.152, 95 % CI: 1.089–1.218). The more advanced the tumor grade was the less likelihood of the tumor being a Luminal A tumor whereby having tumor grades of 3 had a 92 % less risk of being Luminal A tumors (aOR 0.022, 95 % CI: 0.01–0.051).

**Table 6:** Association of predictors of each molecular subtype.

Independent variable	Unadjusted Odds Ratio			Adjusted Odds Ratio		
	OR	95 % CI	P – value	OR	95 % CI	P – value
Luminal A						
Age	1.009	0.998–1.019	0.103	1.011	1.001–1.022	0.055
Tumor size	1.074	1.022–1.129	0.005	1.152	1.089–1.218	0.001
Nottingham tumor grade						
I	Ref					
II	0.167	0.093–0.299	0.001	0.131	0.071–0.239	0.001
III	0.033	0.015–0.071	0.001	0.022	0.022–0.051	0.001
Lymph node involvement						
No	Ref					
Yes	0.791	0.584–1.072	0.131	0.874	0.626–1.221	0.431
Luminal B						
Age	0.991	0.982–1.001	0.054	0.989	0.98–0.998	0.03
Tumor size	1.005	0.961–1.051	0.835	1.06	1.008–1.115	0.023
Nottingham tumor grade						
I	Ref					
II	1.631	0.902–2.946	0.105	1.662	0.908–3.043	0.1
III	0.202	0.098–0.416	0.001	0.217	0.103–0.457	0.001
Lymph node involvement						
No	Ref					
Yes	0.471	0.363–0.611	0.001	0.531	0.4–0.704	0.001
HER2 Enriched						
Age	0.985	0.972–0.998	0.027	0.986	0.973–0.998	0.041
Tumor size	1.101	1.038–1.168	0.001	1.077	1.013–1.146	0.018
Nottingham tumor grade						
I	Ref					
II	3.653	0.910–4.864	0.062	3.69	0.637–4.177	0.087
III	4.765	1.312–7.268	0.026	4.498	1.098–5.632	0.047
Lymph node involvement						
No	Ref					
Yes	1.626	1.048–2.524	0.03	1.318	0.833–2.084	0.238
TNBC						
Age	1.01	1.001–1.019	0.035	1.012	1.002–1.023	0.022
Tumor size	0.873	0.827–0.922	0.001	0.744	0.694–0.798	0.001
Nottingham tumor grade						
I	Ref					
II	3.784	2.031–7.601	0.008	3.398	3.046–6.469	0.02
III	6.105	3.441–8.928	0.001	6.706	30.923–8.004	0.001
Lymph node involvement						
No	Ref					
Yes	2.452	1.792–3.357	0.001	2.325	1.635–3.307	0.001

Luminal B tumors were associated with the age of the patient, tumor size, tumor grade and presence of lymph node involvement. Being older would decrease the likelihood of having Luminal B tumors (aOR 0.989, 95 % CI: 0.98–0.998) hence older women with breast cancer had a 2 % decreased likelihood of having a Luminal B tumor. Patients who had a larger tumor were more likely to have a Luminal B tumor with a 6 % increased likelihood (aOR 1.06, 95 % CI: 1.008–1.115). Tumors that had favorable pathological characteristics were more likely to be Luminal B tumors as the likelihood of having a Luminal B tumor decreased by 79 % when having an advanced tumor (aOR 0.217, 95 % CI: 0.103–0.457) and by almost 50 % in presence of lymph node involvement (aOR 0.531, 95 % CI: 0.4–0.704).

The HER2 enriched tumors were associated with age, tumor size and tumor grade. Similar to Luminal B tumors being older decreased the likelihood of having a HER2 enriched tumor (aOR 0.986, 95 % CI: 0.973–0.998) where as having a larger tumor increased the likelihood by 7 % (aOR 1.077, 95 % CI: 1.013–1.146). However, tumors having an advanced grade at presentation were more than 4 times more likely to have a HER2 enriched tumor (aOR 4.498, 95 % CI: 1.098–5.632).

The TNBC were associated with patients age, tumor size, tumor grade and presence of lymph node involvement. Older women with breast cancer were more likely to have TNBC whereby having an increased age had a weak increased odds of having a TNBC by 1.2 % (aOR 1.012, 95 % CI: 1.002–1.023). As compared to other molecular subtypes having a larger tumor was 25 % less likely to be a TNBC (aOR 0.744, 95 % CI: 0.694–0.798). Having breast cancer with unfavorable pathological characteristics increased the likelihood of having an underlying TNBC whereby having an advanced tumor grade (aOR 6.706, 95 % CI: 3.923–8.004) and presence of lymph node involvement (aOR 2.325, 95 % CI: 1.635–3.307) increased the likelihood of TNBC by 6.7 and 2.3 times respectively.

When molecular subtype outcome was grouped according to only presence of hormonal receptor positivity (that is being a Luminal type tumor), significant factors associated with Luminal type tumors (p-value <0.05) were tumor size, tumor grade and lymph node involvement. These significant variables were run in a binary logistic regression (Table 7) which revealed having a larger tumor (aOR 1.217, 95 % CI: 1.149–1.291) and no lymph node involvement (aOR 0.429, 95 % CI: 0.313–0.589) increased the likelihood of having a Luminal type tumor. Whereas having an advanced tumor grade reduced the risk of having a Luminal type tumor by over 50 % (aOR 0.041, 95 % CI: 0.011–0.019).

**Table 7:** Factors associated with presence of hormonal receptor positivity.

Independent variable	Unadjusted Odds Ratio			Adjusted Odds Ratio		
	OR	95 % CI	P – value	OR	95 % CI	P – value
Tumor size	1.06	1.013–1.109	0.011	1.217	1.149–1.291	0.001
Nottingham tumor grade						
I	Ref					
II	0.78	0.019–0.322	0.001	0.061	0.014–0.252	0.001
III	0.07	0.012–0.028	0.001	0.041	0.011–0.019	0.001
Lymph node involvement						
No	Ref					
Yes	0.387	0.292–0.512	0.001	0.429	0.313–0.589	0.001

## 4 Discussion

To our knowledge this is the largest study of its kind in Tanzania including both a public and private hospital setting. Access to IHC analysis is limited to 3 centres in our country of study, of which 2 centres were included in the study, with lack of availability to IHC services similar to other countries in Sub Saharan Africa [25].

The median age of patients studied was 50 (IQR: 41–61) with majority within the menopausal age group of 40–55 years which is similar to age of presentation of breast cancer in other studies in Western, Eastern and Northern Africa [26,27]. This is younger than women in European countries of Caucasian decent of which majority present in the post-menopausal age group [28]. The most common histological subtype was IDC NST accounting for 89.7 % which was as observed in most studies on breast cancer worldwide accounting for at least three quarters of all histological subtypes [29]. The median tumor size was 5 cm (IQR: 4–7) with more than 70 % having lymph node involvement and such unfavorable pathological characteristics at presentation has been seen in studies all over Africa while women from Europe tend to present with smaller tumors without lymph node involvement [30]. Possible reasons for advanced stage with unfavorable pathological characteristics at presentation in Tanzania have been studied and attributed to poor referral system, lack of screening programs and natural aggressive tumor behavior [31]. The distribution of molecular subtype was not influenced by ethnicity, region of origin nor if they attended a public or private hospital hence generalizations towards the entire study country was made with regards to molecular subtype.

In our study ER and PR positivity was seen in 54.4 % and 34.8 % respectively which tallies with findings in other Eastern and Northern African countries, Kenya and Morrocco respectively, however is more than Western African countries [[14], [15], [16],^18^,^21^]. Expressing hormonal receptor positivity would warrant endocrine therapy with more than 50 % of our study population expressing either or both ER and PR hence 1 of every 2 patients would benefit from drugs such as tamoxifen. Over 50 % of tumors were Luminal tumors with Luminal A accounting for 21.17 % (95 % CI: 18.87–23.47) and Luminal B the most prevalent molecular subtype at 35.75 % (95 % CI: 33.05–38.45). Similar distribution was seen in neighboring East African countries however a significantly less Luminal distribution was noted among Western African countries [21,32]. Despite Luminal breast cancer being the predominant molecular subtype, it was still less as compared to European countries and Caucasian Americans which had a proportion of luminal tumors of up to 70–80 % [33,34].

Luminal tumors have been noted to have better prognosis with more favorable pathological characteristics as also seen in our study whereby these tumors had a better tumor grade and less likelihood of lymph node involvement as compared to HER2 enriched or TNBC [8,35]. Our findings tallied with other studies within Eastern Africa with regards to luminal tumors having favorable pathological characteristics [36]. This was further explored with studies from Northern American countries showing Luminal tumors ultimately had higher rates of survival and lower rates reoccurrence thus now being considered as a factor towards better prognosis [37,38].

The HER2 enriched tumors were seen in 11.86 % (95 % CI: 10.04–13.68) of patients which was comparable to other African countries with most studies showing a range of 10–20 % with similar rates also seen in Western and Asian societies [21,39]. Historically these tumors were associated with poorer prognosis with adverse pathological characteristics most notably lymph node involvement and distant metastasis however an improved survival has now been seen with the use of chemotherapeutic agents such as trastuzumab and lapatinib [40]. As seen with our study HER2 enriched tumors had an increased likelihood for lymph node involvement however agents such as trastuzumab are not actively used in African countries due to cost and availability [41]. Despite trastuzumab being listed as an essential medicine by the World Health Organisation for HER2 positive breast cancer, its high cost renders it inaccessible for the majority with studies in neighboring East African countries concluding it as a drug that was not affordable through government purchasing [42].

It has been established that African American women develop breast cancer at an earlier age with more aggressive histopathological patterns, of note was the tendency of developing TNBC [19]. The predisposition of TNBC was also noted among West African women with some studies showing 50 % up to 70 % of their women with breast cancer as TNBC [27,43]. Due to the high prevalence of TNBC it has been noted women from those regions had less favorable pathological characteristics and worse prognosis when compared to the rest of Africa and Caucasian American women [44]. In our study we had less prevalence of TNBC then our Western African counterparts however shared similarity in the fact that in our group of women who expressed TNBC were more likely to have less favorable pathological characteristics. Studies in Kenya further went on to show that despite prevalence of TNBC among their region was less, they were the subgroup that had a higher likelihood of metastasis and poorer prognosis [36].

It was therefore assumed that women of African ancestry predominantly develop TNBC however studies in Kenya challenged this notion as well as our findings that illustrate Luminal subtype take predominance with TNBC accounting for 31.22 % (95 % CI: 28.61–33.83) only [20,21]. Despite heterogenicity in molecular subtype distribution it is assumed TNBC accounts for 15–20 % of all breast cancers with European and American white women having an even lower distribution of around 10 % [45]. Hence our TNBC distribution is more than European counterparts however we are not a TNBC predominant region as hypothesized previously but rather have a tendency for hormonal receptor expression.

## 5 Conclusion

The most common molecular subtype was Luminal B followed by TNBC, Luminal A and lastly HER2 enriched. We are therefore a not predominant TNBC region however its prevalence is still higher than the average worldwide distribution. Luminal tumors were more common and associated with more favorable pathological characteristics presenting at an earlier age as compared to HER2 enriched and TNBC.

The findings from this study can be used to address peri-surgical cancer care agendas as each subtype has its own algorithm of care and inclination towards prognosis hence resources can be distributed according to prevalence of each subtype. Also, as we are a Luminal predominant region, we recommend the endorsement of endocrine therapy resource availability and encourage the routine use of IHC on all patients with breast cancer.

## Funding

This research did not receive any specific grant from funding agencies in the public, commercial, or not-for-profit sectors. The study design, data collection, analysis, interpretation of results, and writing of this dissertation were carried out by the authors without any financial assistance.

## Ethical approval

The study was performed after ethical approval by the Ethical Review Committee of Aga Khan University (IED/2022/009/fb/016) and Muhimbili National Hospital (MNH/TRCU/Perm/2022/036) in line with the university policies, laws and regulation and ethical guidelines.

## Data sharing statement

The datasets used and analysed during the current study are available with the author. Data will be made available on request.

## CRediT authorship contribution statement

**Allyzain Ismail:** Writing – review & editing, Writing – original draft, Visualization, Validation, Project administration, Methodology, Investigation, Formal analysis, Conceptualization. **Sajida Panjwani:** Writing – review & editing, Visualization, Methodology, Investigation, Conceptualization. **Neelam Ismail:** Writing – review & editing, Supervision, Methodology, Conceptualization. **Caroline Ngimba:** Methodology, Conceptualization. **Innocent Mosha:** Investigation, Data curation. **Philip Adebayo:** Writing – review & editing, Supervision, Methodology, Conceptualization. **Ally Mwanga:** Writing – review & editing, Supervision. **Ali Akbar Zehri:** Writing – review & editing, Supervision. **Aidan Njau:** Writing – review & editing, Supervision, Conceptualization. **Ali Athar:** Writing – review & editing, Visualization, Validation, Supervision, Project administration, Methodology, Investigation, Formal analysis, Data curation, Conceptualization.

## Declaration of competing interest

The authors declare that they have no known competing financial interests or personal relationships that could have appeared to influence the work reported in this paper.

## Figures and Tables

**Appendix I:** Data Collection table

Serial Number	Example 1
MR Number	156-98-82
Region (Location)	Mwanza
Age (Years)	26
Ethnicity	African
Tumour size (Largest dimension in cm)	5
Tumour grade	1
Lymph node involvement (Yes/No)	No
Histological type of breast cancer	Invasive ductal carcinoma
Estrogen receptor % of nuclei stained	40
Estrogen receptor status (Positive/Neg)	Positive
Progesterone receptor % of nuclei stained	35
Progesterone receptor status (Positive/Neg)	Positive
HER receptor score (0,1+,2+,3+)	1+
HER receptor status (Positive/Neg)	Neg
Ki-67 status (%)	10
Molecular subtype	Luminal A

**Figure:**
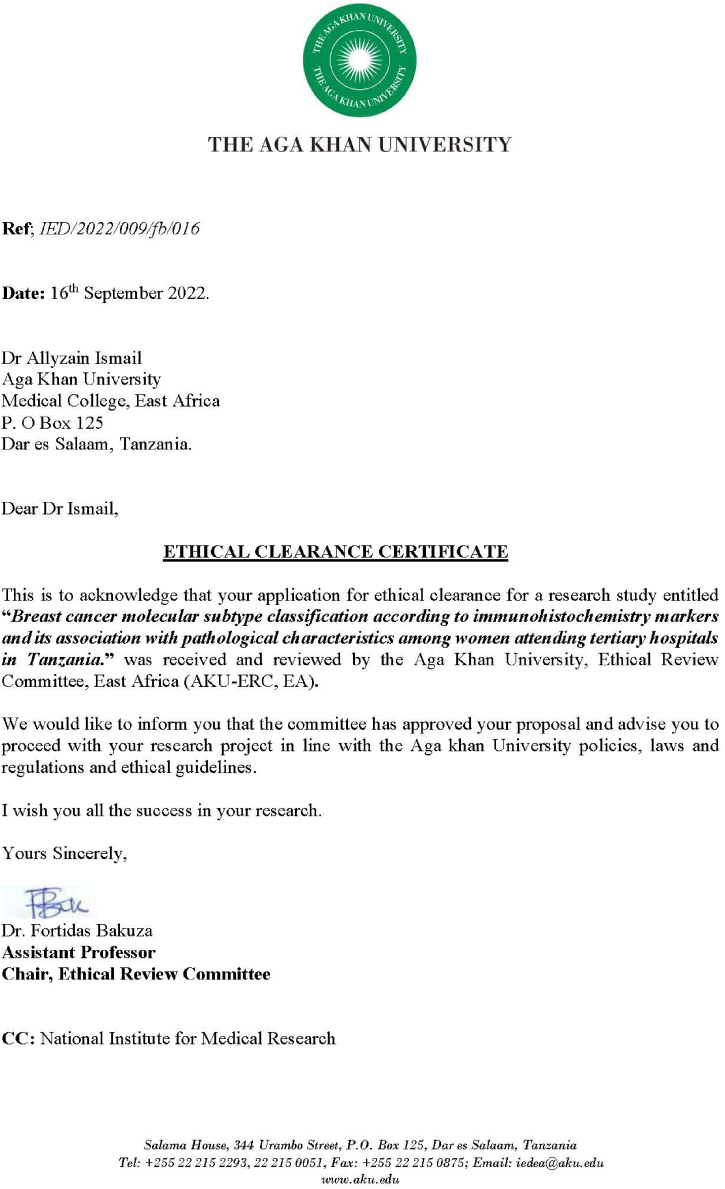


**Figure:**